# Macrophages Help NK Cells to Attack Tumor Cells by Stimulatory NKG2D Ligand but Protect Themselves from NK Killing by Inhibitory Ligand Qa-1

**DOI:** 10.1371/journal.pone.0036928

**Published:** 2012-05-18

**Authors:** Zhixia Zhou, Cai Zhang, Jian Zhang, Zhigang Tian

**Affiliations:** 1 Institute of Immunopharmacology and Immunotherapy, School of Pharmaceutical Sciences, Shandong University, Jinan, Shandong, China; 2 Department of Microbiology and Immunology, School of Life Sciences, University of Science and Technology of China, Hefei, Anhui, China; Centre de Recherche Public de la Santé (CRP-Santé), Luxembourg

## Abstract

Natural killer (NK) cells and their crosstalk with other immune cells are important for innate immunity against tumor. To explore the role of the interaction between NK cells and macrophages in the regulation of anti-tumor activities of NK cells, we here demonstrate that poly I:C-treated macrophages increased NK cell-mediated cytotoxicity against target tumor cells in NKG2D-dependent manner. In addition, IL-15, IL-18, and IFN-β secreted by poly I:C-treated macrophages are also involved in NKG2D expression and NK cell activation. Interestingly, the increase in expression of NKG2D ligands on macrophages induced a highly NK cell-mediated cytotoxicity against tumor cells, but not against macrophages themselves. Notably, a high expression level of Qa-1, a NKG2A ligand, on macrophages may contribute to such protection of macrophages from NK cell-mediated killing. Furthermore, Qa-1 or NKG2A knockdown and Qa-1 antibody blockade caused the macrophages to be sensitive to NK cytolysis. These results suggested that macrophages may activate NK cells to attack tumor by NKG2D recognition whereas macrophages protect themselves from NK lysis *via* preferential expression of Qa-1.

## Introduction

Natural killer (NK) cells are an integral component of the innate immune system and are characterized by their strong cytolytic activity against tumors and virus-infected cells. NK cells also regulate innate and adaptive immune responses through secretion of immunoregulatory cytokines and cell-to-cell contact [1], [2], [3], [4]. NK cells identify susceptible targets via a set of activating or inhibitory receptors that recognize self-protein ligands that are typically up-regulated in transformed or infected cells [1], [2], [5], [6]. The activating receptor NKG2D is the best-characterized receptor expressed by all NK cells and some subsets of NKT cells or T cells. NKG2D could contribute to activation of NK cells via NKG2D-NKG2D ligand interaction. The ligands for NKG2D in mice, including retinoic acid early inducible-1 (RAE-1) proteins (RAE-1α, β, γ, δ, and ε), minor histocompatibility antigen (Ag) H60 and murine UL16-binding protein-like transcript-1 (MULT-1) glycoprotein, are expressed poorly by most normal cells but up-regulated in tumor cells, infected cells or by cells under stress [7], [8], [9]. CD94/NKG2A is one of the major inhibitory receptors in mice, and it recognizes the non-classical major histocompatibility complex (MHC) molecules Qa-1 that is expressed by most cell types. It has been suggested that the Qa-1-CD94?NKG2A interaction is critical for preventing NK cell-mediated killing of mature dendritic cells (DCs) [10], [11]. Therefore, the actions of NK cells are thought to be mediated by the complex interactions between inhibitory and activating signals sent by cell surface receptors following ligation. Moreover, cytokines, such as interleukin (IL)-2, IL-15, IL-12, IL-18 and IL-21, usually produced by other immune cells, and particularly activated antigen-presenting cells (APCs), also play important roles in the regulation of NK cell activity [12], [13].

The crosstalk between NK cells and other cellular lineages has implications in the development of both innate and adaptive immune responses. A great deal of interest and information has emerged with respect to the DC and NK cell crosstalk in contrast to the interactions between NK cells and other innate immune system cells. DCs can activate resting NK cells under conditions involving direct cell-to-cell contact, following stimulation with various pathogens or by Toll-like receptor (TLR) ligands including bacterial lipopolysaccharide (LPS) (an agonist of TLR4) and polyriboinosinic-polyribocytidilic acid (poly I:C) (an agonist of TLR3), leading to the development of dendritic-cell-activated killers (DAKs) [14], [15], [16]. Macrophages are also important effector cells of innate immune responses and can be found distributed throughout the body poised to initiate innate and acquired immune responses. They exert their function by directly sensing a wide variety of pathogen-associated molecules via pattern recognition receptors [17], [18]. Recently, it has been shown that there is also crosstalk between macrophages and NK cells, which exerts important role in antitumor and antiinfection responses [19], [20], [21], [22]. For example, human macrophages treated with LPS induced NK cell cytotoxicity and triggered NK cell cytokine secretion and proliferation. The up-regulation of human NKG2D ligands on LPS-activated macrophages mediated the interaction between NK cells and macrophages [19]. However, it is still unclear how the interaction between NK and macrophage affects NK-mediated cytotoxicity against tumor cells.

In this study, we observed that poly I:C-treated macrophages increased NK cell-mediated cytotoxicity against tumor cells. Interestingly, macrophages themselves were not killed by these activated NK cells. Further results indicated that the preferential expression of Qa-1, the NKG2A ligand, protected macrophages from cytolysis of NK cells.

## Materials and Methods

### Mice and Cell Lines

BALB/c and C57BL/6 mice (6 to 8 weeks-old) were purchased from the Experimental Animal Center of Beijing University (Beijing, China) and maintained under specific pathogen-free conditions. All animal experiments and protocols were approved by the Committee on the Ethics of Animal Experiments of the Shandong University. The murine macrophage-like cell line RAW264.7, the murine lymphoma cell line YAC-1, the murine colon adenocarcinoma cell line MCA-38, the murine hepatocellular carcinoma cell line Hepa1-6, and the murine ascitic hepatoma cell line and H22 were grown at 37°C with 5% CO_2_ in RPMI 1640 medium (Invitrogen, Carlsbad, CA) supplemented with 100 U/ml penicillin, 100 µg/ml streptomycin and 10% fetal bovine serum (FBS).

### Activation of Macrophages in vitro

Resident peritoneal macrophages from wild-type mice were harvested by peritoneal lavage with 5 ml of cold phosphate-buffered saline (PBS). Cells were centrifuged (350×g, 5 min) at room temperature and allowed to adhere to 6-well plates for 2 h (nonadherent cells were removed by washing with PBS). The adherent cells (primary macrophages) or RAW264.7 cells were incubated with medium or various concentrations of poly I:C (Sigma, St. Louis, MO). To investigate the contact dependence of the interaction, macrophages and NK cells were separated by a membrane (0.4-µm pore size) in Transwell plates (Costar; Corning) in some experiments.

### Real Time (RT)-PCR Analysis

Total RNA was isolated from cells using TRIzol as described by the manufacturer (Invitrogen). cDNA was generated with Oligo dT primer using M-MLV reverse transcriptase (Promega, Madison, WI) according to the manufacturer’s protocol. PCR primers for detecting the indicated mRNA (Sangon Ltd., Shanghai, China) are described in Table 1. Triplicate 20 µl PCR reactions were carried out using SYBR Green Supermix (BioRad) incubated for 10 min at 95°C followed by 45 cycles consisting of 95°C for 15 s, 60°C for 15 s and 72°C for 15 s. The levels of mRNA were normalized to that of β-actin.

**Table 1:** Sequences of primer used for real-time PCR assay.

Target sequence	Sequence (5′-3′)	size (bp)
β-actin	R: AGAGGGAAATCGTGCGTGACF: CAATAGTGATGACCTGGCCGT	139
RAE-1	R: ATCAACTTCCCCGCTTCCAF: AGATATGAAGATGAGTCCCACAGAGATA	79
H60	R: GAGCCACCAGCAAGAGCAAF: CCAGTATGGTCCCCAGATAGCT	75
MULT-1	R: TTCACATAGTGCAGGAGACTAACACAF: ACTGGCCACACACCTCAGC	69
Qa-1a	R: GCGGTATTTCCACACTGCCAF: TCTGTGAGGCAAAGTCAGTC	80
Qa-1b	R: CCTGGACCGCGAATGACATAF: CACCACAGCTCCAAGGATGAT	514
NKG2D	R: ACGTTTCAGCCAGTATTGTGCF: GGAAGCTTGGCTCTGGTTC	132
TRAIL	R: CCCTGCTTGCAGGTTAAGAGF: G GCCTAAGGTCTTTCCATCC	240
FasL	R: AAGAAGGACCACAACACAAATCTGF: CCCTGTTAAATGGGCCACACT	233
Perforin	R: GAGAAGACCTATCAGGACCAF: AGCCTGTGGTAAGCATG	166
TLR3	R: TCTGGAAACGCGCAAACCF: GCCGTTGGACTCTAAATTCAAGAT	85
TLR4	R: AGAAATTCCTGCAGTGGGTCAF: TCTCTACAGGTGTTGCACATGTCA	84
IL-18	R: GCCATGTCAGAAGACTCTTGCGTCF: GTACAGTGAAGTCGGCCAAAGTTGTC	122
IL-15	R: TTAACTGAGGCTGGCATTCATGTCTTCF: CAGTTCATTGCAGTAACTTTGCAACTG	194
IL-12p35	R:CATCGATGAGCTGATGCAGTF: CAGATAGCCCATCACCCTGT	163
IL-12p40	R:TGGAAGCACGGCAGCAGAATAAATF:TGCGCTGGATTCGAACAAAGAACT	202
IFN-β	R:CCATCATGAACAACAGGTGGATF:GAGAGGGCTGTGGTGGAGAA	67
IFN-γ	R: TGCATCTTGGCTTTGCAGCTCTTCCTCATGGCF: TGGACCTGTGGGTTGTTGACCTCAAACTTGGC	194
IFN-α	R: TCTGTGCTTTCCTCGTGATGF: TTGAGCCTTCTGGATCTGCT	215

### NK Cell Isolation

Splenocytes were incubated with anti-CD49b (DX-5, isotype: rat IgM; clone: DX-5) microBeads (Miltenyi Biotec, Auburn, CA) and DX-5^+^ cells (NK cells) were isolated using MACS Separation Columns (Milteny Biotec, Auburn, CA) according to the manufacturer’s instructions. CD49b^+^ NK cells were identified by flow cytometry using a FITC-conjugated anti-CD49b antibody (Ab) (isotype: rat IgG; clone: HMa2; eBioscience) and the purity was confirmed to be ≥85%. For *in vitro* analyses, DX-5-sorted cells were cultured in RPMI 1640 supplemented with 100 U/ml human IL-2 (Peprotech, London, UK) to maintain the activity of NK cells.

### Cytotoxicity Assays

NK cell-mediated cytotoxicity was determined by ^51^Cr release assay (11). Prior to incubation with ^51^Cr -labeled target cells, NK cells were pre-incubated with poly I:C-untreated or -treated macrophages. Target cells were seeded into 96 well round-bottom plates at 2×10^4^ cells per well. Effector cells were added to target cells at effector/target (E/T) ratios of 50∶1, 25∶1 and 5∶1 separately. For NKG2D or Qa-1 blocking assays, NK cells or macrophages respectively were treated with 10 µg/ml purified anti-mouse NKG2D- or Qa-1 neutralizing mAb (R&D Systems, Minneapolis, MN) for 4 h before being used. For IL-15 or IFN-β blocking assays, NK cells were cultured with poly I:C-untreated or -treated macrophages in the presence of 10 µg/ml anti-IL-15, anti-IFN-β, or isotype control mAbs, and then NK cells were added to target cells. The cell mixtures were then incubated for 4 h. And the culture supernatants were assayed for ^51^Cr release using a gamma radiation counter. Percentage of specific lysis was determined by the formula: (sample release - spontaneous release)/(maximum release-spontaneous release) ×100%.

**Figure 1:**
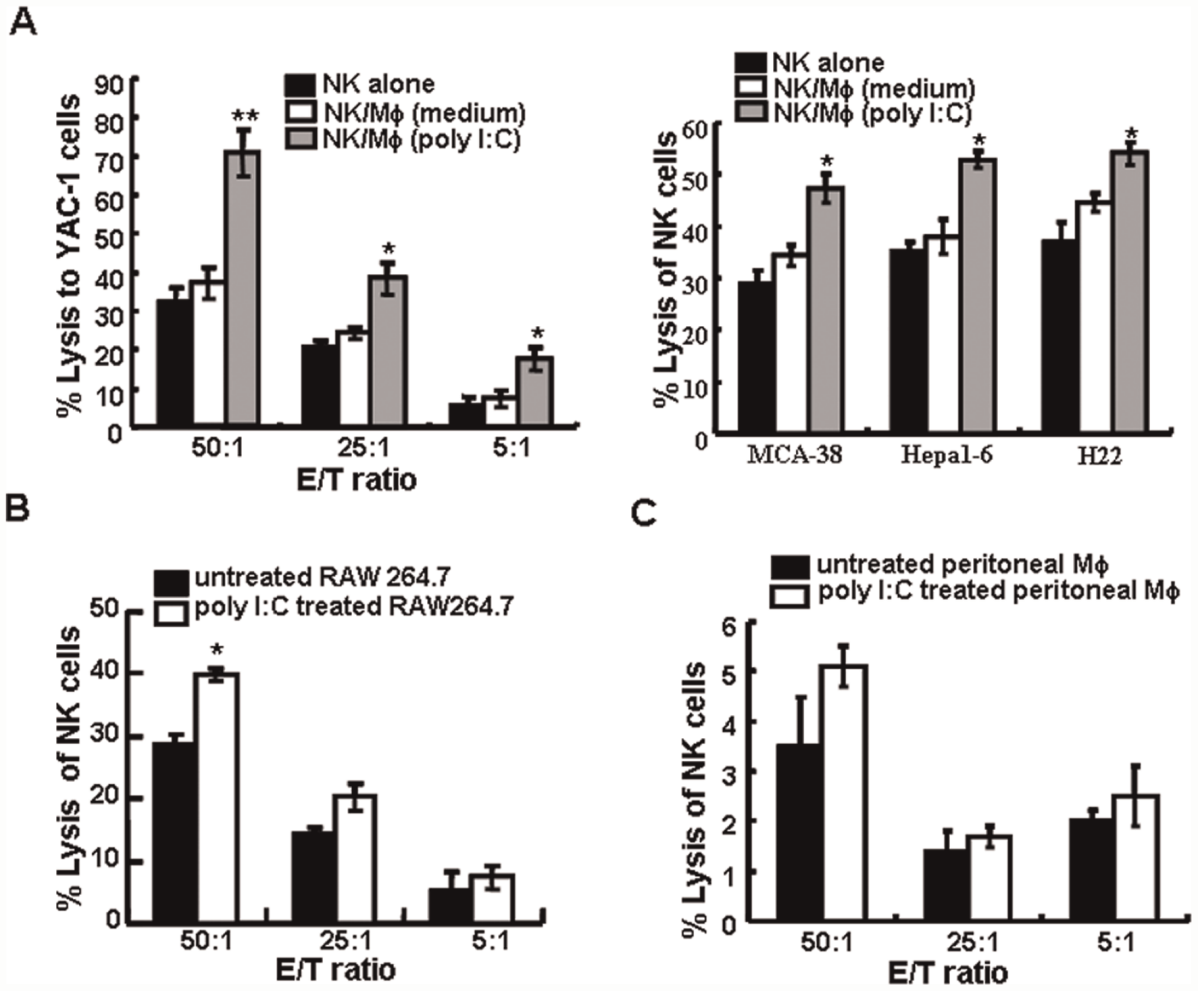
Murine macrophages increase NK cell cytotoxicity to susceptible target cells. Freshly purified splenic NK cells were first co-cultured with untreated or poly I:C-pretreated macrophages for 24 h at an NK/macrophage ratio of 5∶1, and then separated by vibrating gently to be used as effector cells against susceptible target cells. NK cell-mediated lysis against YAC-1 cells, Hepa1-6 cells, MCA38 cells, H22 cells (A), RAW264.7 cells (B) or peritoneal Mφ (C) was assessed by the ^51^Cr release assay at various E:T rations. Data are expressed as the mean ± SD from at least three separate experiments. *p<0.05, **p<0.01 versus untreated cells using the paired Student’s test.

### Flow Cytometric Analysis

For cell surface staining, cells were harvested, blocked with anti-FcγR mAb, and stained with the labeled mAbs at 4°C for 45 min. For intracellular cytokine staining, cells were cultured in RPMI 1640 containing 10% FCS, and treated with monensin (Sigma) for 4 h to inhibit extracellular secretion of cytokines. IFN-γ was determined by intracellular flow cytometry. The antibodies used were as followed: carboxyfluorescein-conjugated RAE-1, FITC-conjugated MULT-1, PE-conjugated H60 (R&D Systems), anti-mouse NKG2D mAb (R&D System), PE-conjugated Ab to CD68, CD69 or IFN-γ (R&D System), FITC-conjugated affinity-purified goat anti-rat IgG (ZSGB-BIO, China) or isotype controls (eBioscience, San Diego, CA, USA). All mAbs were used at a final concentration of 10 µg/mL. All stained cells were analyzed using a flow cytometer (FACScalibur, USA), and the data were processed with WinMDI 2.9 software (Scripps Research Institute).

### ELISA for Cytokine Detection

The level of IFN-γ in cell culture supernatants was detected by commercial enzyme-linked immunosorbent assay (ELISA) kits (R&D Systems), following the manufacturers’ instructions. The levels of IL-12, IL-15 and IFN-β were measured using ELISA kits (Adlitteram Diagnostic, USA).

### RNA Interference

Transfections of siRNAs targeting Qa-1 or TLR3 were carried out using lipofectamine 2000 (Invitrogen, Carlsbad, CA) at a final small interfering (si) RNA concentration of 100 nM according to the manufacturer’s instructions. The sense siRNA strands used in this study were as follows: Qa-1, GAAGAGGAGGAGACACAUA and TLR3, GAGCATCAATCTAGGACTGAA. All siRNAs were purchased from Ribobio (Guangzhou, China).

### Statistical Analysis

In each experiment, the results were expressed as the mean ± SD. Student's t test was used to compare the differences between two different groups, and *P*<0.05 was considered statistically significant.

**Figure 2:**
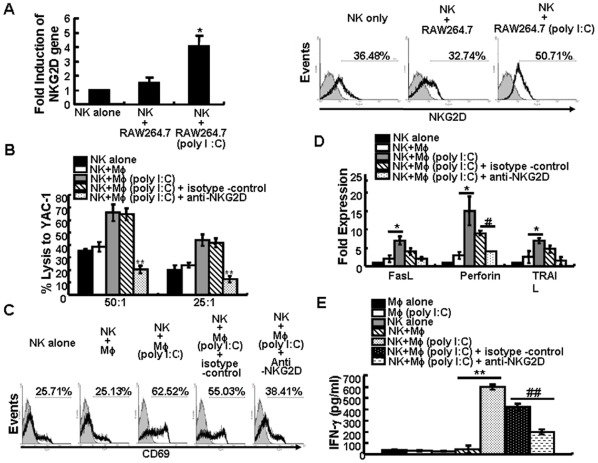
Poly I:C-treated macrophages enhanced NKG2D expression and NK cell function. Freshly isolated splenic NK cells were co-cultured with RAW264.7 cells that were treated with 0 or 100 µg/ml of poly I:C for 24 h. NK cells were then isolated and NKG2D expression was determined by RT-PCR and flow cytometry (A). Data are expressed as the fold change in mRNA expression normalized to untreated cells or as the percentage of positively-stained cells. * p<0.05 versus untreated cells using the paired Student’s test. The isolated NK cells from co-culture with macrophages were co-incubated with saturating amounts of anti-NKG2D mAb or isotype control, washed and used as effector cells against YAC-1 targets (B). The expression of CD69 (C), cytotoxic effector molecules FasL, TRAIL, and perforin (D), and seceretion of IFN-γ (E) were determined by flow cytometry, RT-PCR, and ELISA separately. Data are expressed as the mean ± SD from at least three separate experiments. *p<0.05, **p<0.01 compared with poly I:C-untreated RAW264.7 group; ^#^ p<0.05, ^##^ p<0.01 compared with isotype control group.

## Results

### Poly I:C-treated Macrophages Increase NK Cell-mediated Cytotoxicity to Target Tumor Cells, but not to Macrophages

To investigate whether poly I:C-treated macrophages affect NK cell-mediated cytotoxicity, we used ^51^Cr -labeled tumors as targets. The splenic CD49^+^ NK cells were isolated and purified from BALB/c or C57BL/6 mice. The purity (85%–92%) was confirmed by flow cytometry analysis (data not shown). These splenic NK cells were co-cultured with poly I:C-treated RAW264.7 cells for 24 h at NK/macrophage ratios of 5∶1, and then separated to be used as effector cells against tumor cells. The purity of splenic NK cells isolated from macrophages was greater than 90% (Fig. S1). NK cytotoxicity against YAC-1 cells, Hepa1-6 cells, MCA38 cells or H22 cells was dramatically increased when NK cells were pre-incubated with poly I:C-treated macrophages (Fig. 1A). Among these target cells, YAC-1 was most sensitive to NK cells isolated from poly I:C-treated macrophages (p<0.05 at E:T ration of 5∶1 and 25∶1, and p<0.01 at E:T ration of 50∶1, respectively, compared with that of NK cells isolated from untreated macrophages). And we also found that compared with untreated NK cells, exposure to untreated macrophages did not result in an increase of NK cells cytotoxicity against both type of target cells (Fig. 1A). These data suggested that poly I:C-treated macrophages significantly enhanced NK cell-mediated cytotoxicity to tumor cells.

Interestingly, no statistically difference was found in NK cells cytotoxicity to resting RAW264.7 cell line, when comparing poly I:C-treated macrophages incubated NK cells with untreated macrophages incubated NK cells or untreated NK cells (data not shown). We further tested whether poly I:C-treated macrophages enhance the cytolytic capacity of NK cells against poly I:C-treated macrophages themselves. We found that NK cells isolated from poly I:C-treated RAW264.7 cells exerted slightly higher cytotoxicity against poly I:C-treated RAW264.7 cells than that against untreated RAW264.7 cells only at the E:T ratio of 50∶1, with the cytotoxicity increased from 28.8±1.93% to 39.7±1.1% (p<0.05) (Fig. 1B). Neither untreated nor poly I:C-treated peritoneal macrophages were lysed by activated autologous NK cells (Fig. 1C).

**Figure 3:**
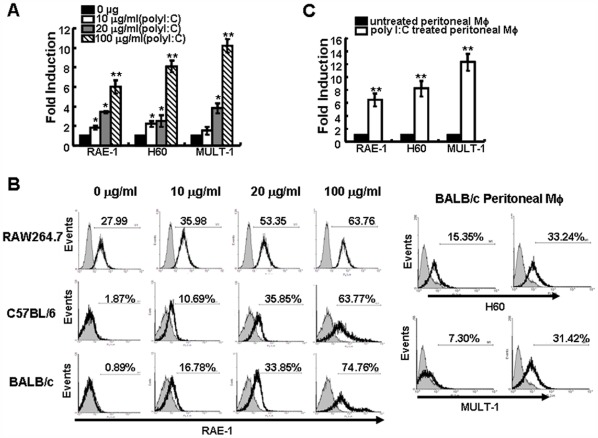
Up-regulation of NKG2D ligands on poly I:C-treated murine macrophages. Murine macrophage RAW264.7 cells were cultured for 24 h with 0, 10, 20 or 100 µg/ml poly I:C and analyzed by RT-PCR or FACS. The data are expressed as fold induction of RAE-1, H60 or MULT-1 mRNA (A) or the percentage of positive RAE-1 staining using monoclonal pan-RAE-1 antibodies (B). Resident peritoneal macrophages from BALB/c or C57BL/6 mice were treated with either 0 or 100 µg/ml poly I:C for 24 h and NKG2D ligand transcripts and surface expression were analyzed by real-time PCR (C) or FACS (B). Data shown are expressed as the mean ± SD from at least three separate experiments. *p<0.05, **p<0.01 compared with untreated cells.

### NK Cell-mediated Target Cell Lysis is NKG2D-dependent

Since NKG2D is an activating receptor for NK cell activation, we determined NKG2D expression on NK cells following exposure to poly I:C-treated macrophages. The splenic NK cells were co-cultured with untreated or poly I:C-treated RAW264.7 cells for 24 h, and NKG2D expression was then analyzed by RT-PCR and flow cytometry. As shown in Fig. 2A, NKG2D expression was markedly increased on splenic NK cells following exposure to poly I:C-treated RAW264.7 cells at both the mRNA and protein levels. We also examined expression of other activating receptors, such as DNAM-1 and NKp46, on NK cells, but found no significant difference on the surface of NK cells co-cultured with untreated or poly I:C-treated macrophages (data not shown). To determine the relative contributions of NKG2D expression on NK cell-mediated lysis, splenic NK cells were first co-cultured with poly I:C-treated RAW264.7 cells for 12 h at an NK/macrophage ratio of 5∶1, and were then co-incubated with saturating amounts of anti-NKG2D mAb or isotype control. NK cells were then washed and used as effector cells against YAC-1 targets. As demonstrated in Fig. 2B, anti-NKG2D mAbs significantly attenuated the NK cell-mediated lysis of YAC-1 cells at every E:T ratio examined (p<0.05, compared with isotype control or without antibody incubation), suggesting that the increased NK cell-mediated YAC-1 lysis was dependent on the activating receptor NKG2D.

The activation status of NK cells following co-culture with poly I:C-treated macrophages was further examined by measuring CD69 expression on NK cells which was significantly up-regulated with the percentage of CD69^+^ NK cells increased from 25.71% to 62.52% (Fig. 2C). In addition, the cytotoxic-related molecules, TNF-related apoptosis-inducing ligand (TRAIL), perforin, and FasL were significantly up-regulated following incubation with poly I:C-treated macrophages (Fig. 2D *)*. And the level of IFN-γ in NK cell supernatants increased significantly after co-cultured with poly I:C-treated macrophages for 12 h (Fig. 2E). To investigate whether NKG2D contributed to the activation status of NK cells, we added saturating amounts of anti-NKG2D mAb or isotype control when incubation of NK cells and poly I:C-treated macrophages. We found that blockade of NKG2D alleviated the expression of CD69 (Fig. 2C), perforin, FasL and TRAIL on NK cells (Fig. 2D), and reduced the level of IFN-γ in the supernatants (Fig. 2E). These experiments demonstrated that poly I:C-treated macrophages activated NK cells by up-regulating NK cell lysis-associated molecules and NKG2D surface expression which correlated with significantly enhanced target cell lysis.

**Figure 4:**
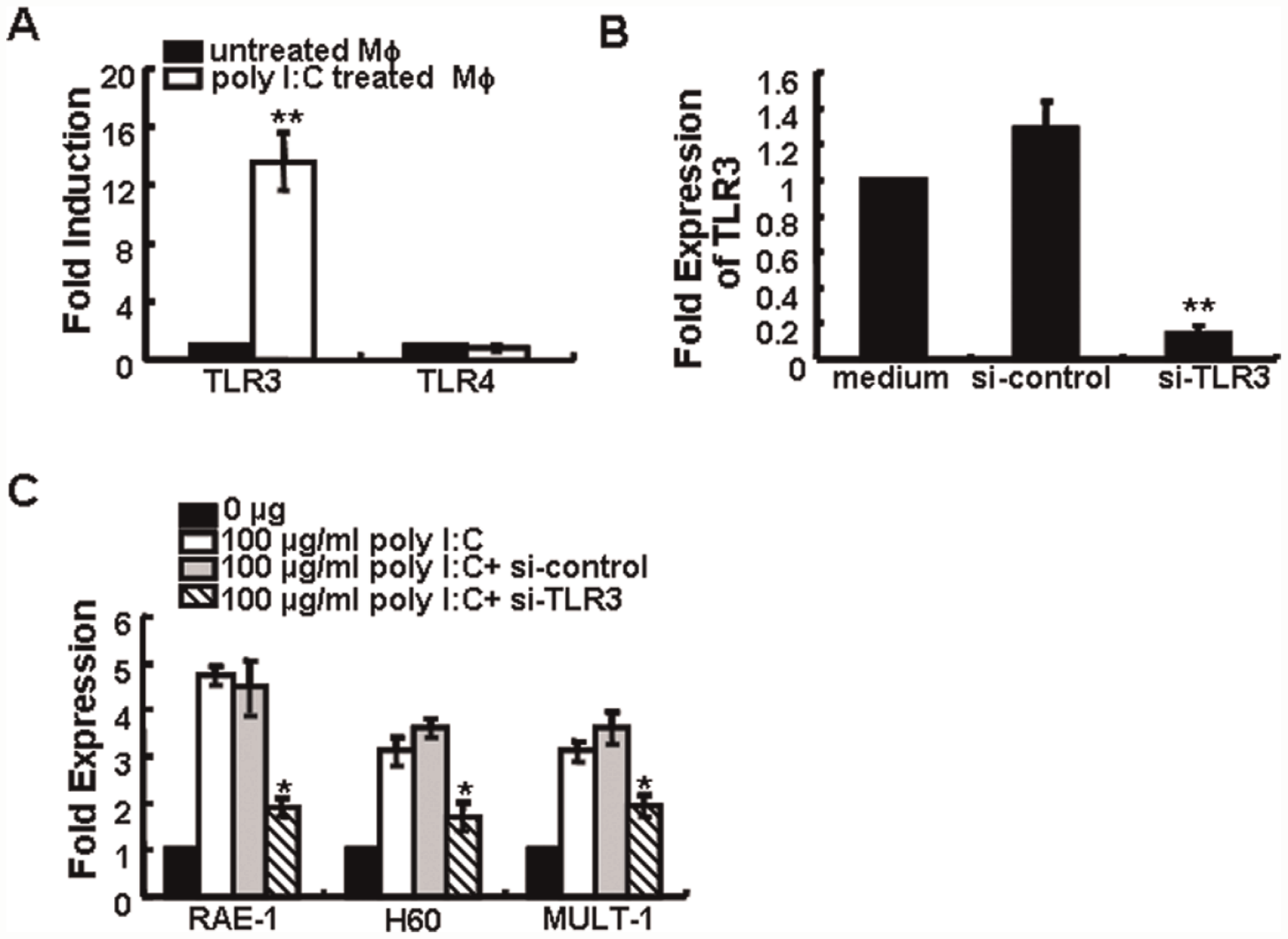
TLR3 is necessary for poly I:C-mediated up-regulation of NKG2D ligands by macrophages. Expression of TLR3 and TLR4 by RAW264.7 cells stimulated with 100 µg/ml poly I:C for 24 h was analyzed by RT-PCR (A). TLR3 mRNA levels of RAW264.7 cells were measured following incubation in media only or following treatment with si-TLR3 or siRNA control (si-control) for 24 h (B). The data are expressed as the folds ± SD from at least three separate experiments. *p<0.05, **p<0.01 compared with the untreated group. RAW264.7 cells first transfected with si-TLR3 or si-control were stimulated with 0 or 100 µg/ml poly I:C for 24 h and the transcripts of the NKG2D ligands RAE-1, H60, and MULT-1 were detected by RT-PCR (C). The data are expressed as the mean induction folds ± SD over untreated cells from at least three separate experiments. *p<0.05, compared with si-control group.

### Poly I:C up-regulates Expression of NKG2D Ligands on Macrophages

Because the interactions between NKG2D and its ligands could mediate NK cells activation, we attempted to determine the changes in the levels of the NKG2D ligands RAE-1, H60 and MULT-1 on RAW264.7 cells following stimulation with poly I:C for 24 h at 0, 10, 20, or 100 µg/ml by RT-PCR. The results showed that the transcripts for these three NKG2D ligands were significantly elevated following poly I:C treatment in a dose-dependent manner (p<0.05 with 10 or 20 µg/ml poly I:C incubation and p<0.01 with 100 µg/ml poly I:C incubation when compared with untreated cells) (Fig. 3A) and a dose-dependent increase in RAE-1 expression was also detected by flow cytometry (Fig. 3B). In addition, resident peritoneal macrophages harvested from BALB/c and C57BL/6 mice were treated with either 0 or 100 µg/ml poly I:C for 24 h and examined by RT-PCR for RAE-1, H60 and MULT-1 expression. Compared with untreated cells, incubation with poly I:C significantly increased transcript levels for each NKG2D ligand examined (p<0.01) (Fig. 3C). Then ligand surface expression paralleled the transcript levels was detected. Flow cytometry analysis showed a dose-dependent increase for RAE-1 expression from both BALB/c and C57BL/6 mice over a 24 h period (Fig. 3B). The expression of H60 and MULT-1 on primary peritoneal macrophages also augmented (Fig. 3B). These results demonstrated that poly I:C could up-regulate NKG2D ligand expression on murine macrophages in a dose-dependent manner.

**Figure 5:**
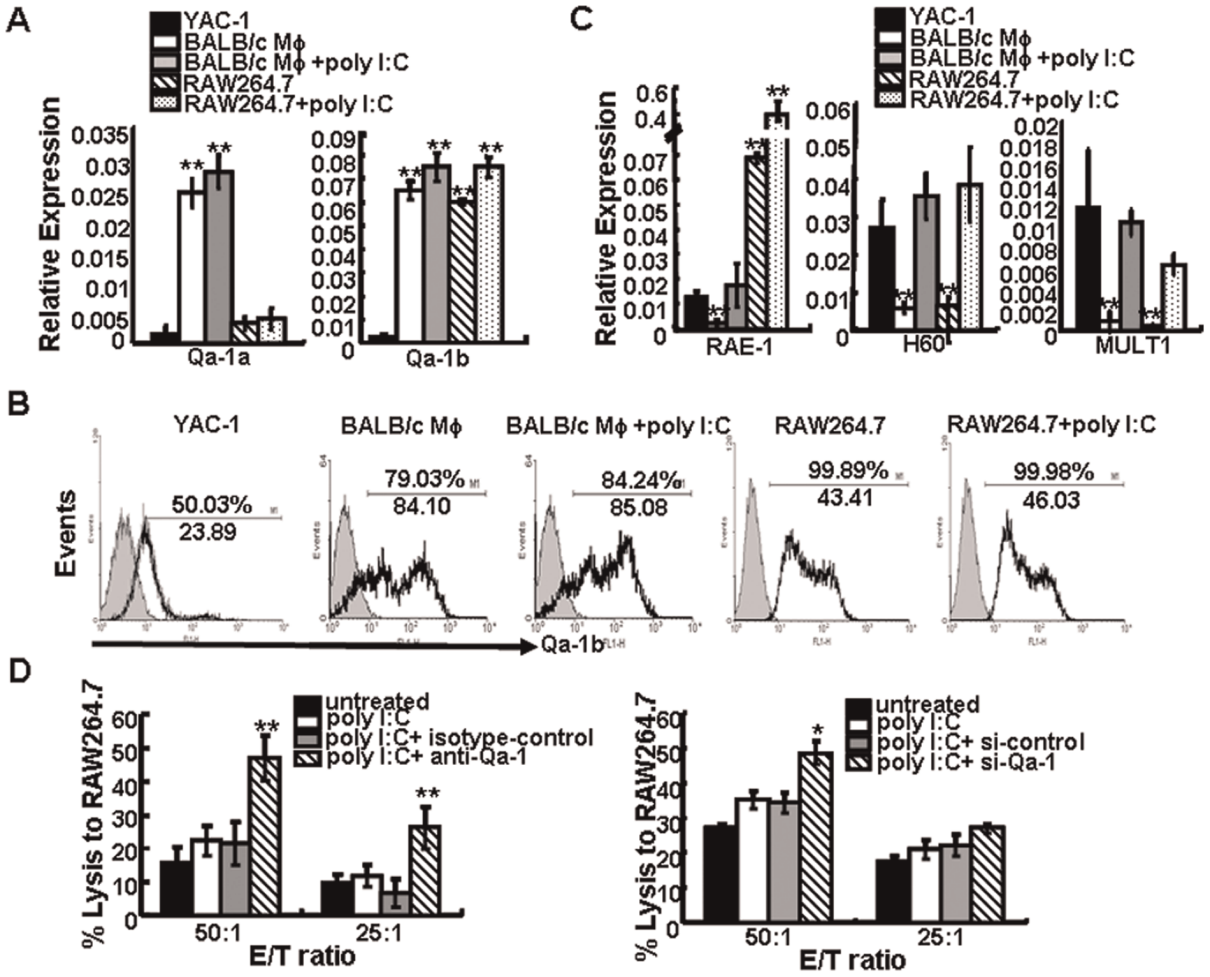
Qa-1 contributes to the protection of macrophages from NK cell-mediated lysis. Changes in mRNA and protein levels of the NKG2A ligands Qa-1a and Qa-1b (A,B), and the NKG2D ligands RAE-1, H60, and MULT-1 (C) in YAC-1 cells, macrophages (Mφ) or RAW264.7 cells were detected by RT-PCR or FACS. Data are expressed as relative expression from at least three separate experiments. *p<0.05, **p<0.01 versus YAC-1 mRNA levels using the paired Student’s test. RAW264.7 cells first stimulated with 100 µg/ml poly I:C for 24 h and then co-incubated with saturating amounts of anti-Qa-1 mAb or isotype control or transfected with si-Qa-1 or si-control, were then used as target cells. The cytolytic activity of NK cells stimulated with poly I:C-treated macrophages was assessed at various E:T rations (D). Data are expressed as the mean ± SD from at least three separate experiments. *p<0.05, **p<0.01 compared with the isotype control or si-control group.

**Figure 6:**
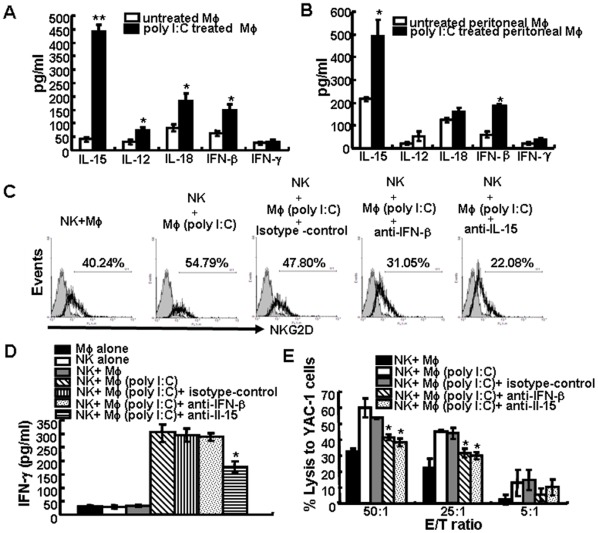
Cytokines secreted by poly I:C-treated macrophages play critical roles in NK cell activation. The secretion levels of IL-12, IL-15, IL-18, IFN-β, or IFN-γ in the supernatants of RAW264.7 cells (A) or resident peritoneal macrophages from BALB/c mice (B) that were cultured with 0 or 100 µg/ml poly I:C for 24 h were analyzed by ELISA. The data are expressed as the mean ± SD from at least three separate experiments. *p<0.05, **p<0.01 compared with the untreated RAW264.7 group. NK cells were incubated with poly I:C-pretreated RAW264.7 cells in the presence of 10 µg/ml anti-IL-15 mAb, anti-IFN-β mAb or isotype control separately for another 12 h, and the expression of NKG2D were determined by flow cytometry (C). Levels of IFN-γ in the supernatants were determined by ELISA (D). NK cell-mediated lysis of YAC-1 cells was assessed by first culturing NK cells with poly I:C-treated RAW264.7 cells (Mφ) in the presence or absence of anti-IL-15 mAb, anti-IFN-β mAb or isotype control, respectively, and then NK cells were isolated and added to target cells (E). Data are expressed as the mean ± SD from at least three separate experiments. *p<0.05 compared with the isotype control group.

**Figure 7:**
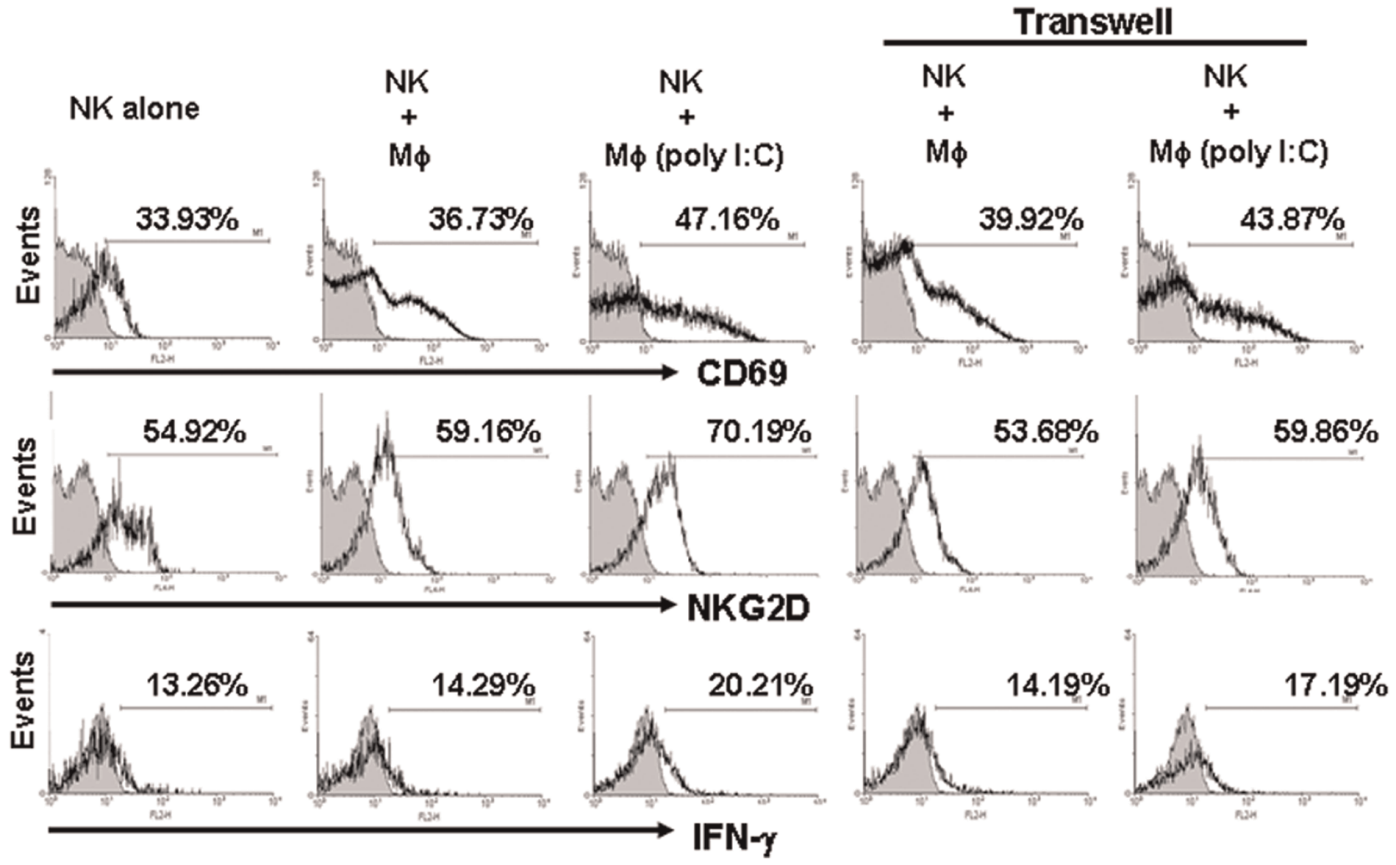
Both cell-to-cell contact and cytokines were involved in the crosstalk between NK cells and macrophages. Freshly isolated murine NK cells were co-cultured with poly I:C-stimulated primary macrophages separated by a transwell for 24 h. Then NK cells were stained with antibodies for cell-surface CD69 and NKG2D, and for intracellular IFN-γ. The numbers of CD69- or NKG2D-positive as well as IFN-γ-secreting NK cells were determined by flow cytometry. Data are expressed as the percentage of positively-stained cells. The data are representatives from at least three separate experiments.

### Poly I:C-induced Up-regulation of NKG2D Ligands is Dependent on TLR3

Poly I:C can bind to and up-regulate TLR3 expression on some tumor cells and enhances cycloheximide-induced apoptosis via a TLR3 pathway [23]. We also measured whether the expression of NKG2D ligands on murine macrophages would be dependent on TLR activation. Poly I:C treatment augmented the expression of TLR3 mRNA of RAW264.7 (Fig. 4A) and peritoneal macrophage (data not shown), but not TLR4. When TLR3 expression on RAW264.7 cells was silenced by TLR3 specific siRNA (Fig. 4B), poly I:C-mediated up-regulation of the three NKG2D ligands was abrogated significantly (Fig. 4C), suggesting that the up-regulation of NKG2D ligands on macrophages by poly I:C treatment was TLR3-dependent.

### Qa-1 Contributes to the Protection of Macrophages from NK Cell-mediated Cytolysis

It has been documented that NK cell activation is tightly regulated by signals from an array of cell-surface inhibitory and activating receptors that interact with target cell ligands *e.g.*, signals transmitted by inhibitory receptors for MHC class I molecules inhibited NK cell-mediated cytolysis [1], [2]. Recent studies have demonstrated that Qa-1b is critically involved in regulating IFN-γ synthesis by NK cells as a result of interactions with the CD94/NKG2A inhibitory receptors that may protect DC from NK cell-mediated cytolysis even in the absence of classical MHC class I molecules [11], [24]. To address whether Qa-1 was also critical in protecting murine macrophages from NK cell-mediated killing, we first compared the expression level of Qa-1 mRNA in poly I:C-treated or untreated macrophages (RAW264.7 cells and peritoneal macrophages freshly isolated from BALB/c mice) and found no significant changes in the Qa-1a and Qa-1b transcript levels following poly I:C stimulation, but the levels of Qa-1a in poly I:C-treated or untreated primary macrophages are much higher than that in YAC-1 cells by 16.7±1.98 folds and 16.9±0.97 folds separately, and the levels of Qa-1b in primary macrophages are higher by about 10 folds (Fig. 5A). Even for RAW264.7 cells, Qa-1a expression is higher than that in YAC-1 cells by 1.78±0.31 and 2.17±0.42 folds, and Qa-1b expression is higher by 9.75±0.43 and 11.95±1.45 folds, respectively (Fig. 5A). Similar results were obtained from the total Qa-1b expression in the three cell types by flow cytometry (Fig. 5B). We also compared the transcription levels of NKG2D ligands in the three cell types and found that the expression of three NKG2D ligands, RAE-1, H60 and MULT-1, was lower in murine primary macrophages than in YAC-1 cells. After treatment with poly I:C, the levels were equivalent with that in YAC-1 cells. As for RAW264.7 cells and YAC-1 cells, the expression of NKG2D ligands was nearly at the same level, except that RAE-1 expression was increased significantly after poly I:C stimulation (Fig. 5C). These results suggested that the expression of inhibitory ligands on macrophages was predominant than that of activating ligands if compared with YAC-1 cells.

To further investigate whether Qa-1 expression contributed to the protection of murine macrophages from NK cell-mediated cytotoxicity, we first incubated RAW264.7 cells with saturating amounts of anti-Qa-1b mAb to block the interaction of Qa-1 with its receptor CD94/NKG2A. We found that blockade of Qa-1 significantly enhanced the cytotoxicity of NK cells stimulated with poly I:C-treated RAW264.7 cells against poly I:C-treated RAW264.7 cell line (Fig. 5D). We further used siRNA to knock down the Qa-1 expression of RAW264.7 cells. As shown in Fig. S2A, the Qa-1 mRNA levels were significantly reduced after Qa-1 siRNA treatment, and NK cell-mediated cytolysis of poly I:C-treated Qa-1-siRNA-silenced RAW264.7 cells was significantly increased at the highest E:T ratio (50∶1) (p<0.05) (Fig. 5D).

In addition, we investigated the NKG2A molecule expression on NK cells and found no significant difference on the surface of NK cells when co-cultured with untreated or poly I:C-treated macrophages (Fig. S2B). Next, splenic CD49^+^ NK cells were treated with NKG2A-siRNA or control-siRNA before incubation with RAW264.7 cells. We found that silence of NKG2A on NK cells enhanced the NK cell-mediated lysis of macrophages (Fig. S2C and Fig. S2D). These results suggested that the interaction of Qa-1 and NKG2A plays a critical role in protection of murine macrophages from NK cell-mediated lysis even when NKG2D ligands were up-regulated on macrophages. Although we can not rule out the role of other inhibitory receptors, the data from Qa-1 knockdown and blockade of Qa-1 and NKG2A did supported the idea that Qa-1 plays major roles in protection of macrophages from NK lysis.

### Poly I:C-stimulated Production of Cytokines by Macrophages also Play a Critical Role in NK Cell Activation

It has been documented that poly I:C stimulation induced the production of several cytokines by macrophages or DCs e.g., IFN-β, IL-12, and IL-15 [25], [26], [27]. We next investigated whether cytokines produced by poly I:C-treated macrophages were involved in NK cells activation. First, changes in the protein levels of IL-12, IL-18, IL-15, IFN-β and IFN-γ were detected by ELISA in the supernatants of RAW264.7 cells and peritoneal macrophages. The results showed that poly I:C stimulation did not affect IFN-γ production, but the secretion levels of IL-15, IFN-β, IL-18 and IL-12 were markedly increased in RAW264.7 cells (Fig. 6A). High level of IL-15 and IFN-β were also found in BALB/c peritoneal macrophages after poly I:C stimulation (Fig. 6B). To investigate whether these two cytokines contributed to the activation status of NK cells, NK cells were cultured with poly I:C-untreated or -treated macrophages in the presence of 10 µg/ml of anti-IL-15 or anti-IFN-β blocking mAb, or isotype control mAbs. Blocking either IL-15 or IFN-β decreased expression of NKG2D (Fig. 6C) and CD69 (data not shown) on NK cells significantly. The secretion of IFN-γ by NK cells was also suppressed by blockade with IL-15 mAb, although it was not impaired by IFN-β neutralization (Fig. 6D). Moreover, with blockade of either of the two cytokines, NK cell-mediated lysis against YAC-1 cells was significantly attenuated (Fig. 6E). These data suggested that cytokines produced by poly I:C-stimulated macrophages, also play critical role in NK cell activation. We further use transwell culture to prohibit cell-to-cell contact between NK cells and macrophages. As shown in Fig. 7, poly I:C-stimulated primary macrophages still promote the expression of CD69 and NKG2D, as well as production of IFN-γ by NK cells, although the up-regulation effect is not as strong as that in direct contact coculture. These finding indicated that both cell-to-cell contact and cytokines secreted by poly I:C-stimulated macrophages play major roles in NK cell activation.

## Discussion

NK cells are bone marrow (BM)-derived lymphocytes involved in immune recognition of infected cells and tumors. They are not only important players of innate effector responses, but also participate in the initiation and development of antigen-specific responses. The presence of both activating and inhibitory receptors on the surface of NK cells act collaboratively with cytokines to regulate NK cell functions [1], [2], [3], [4]. The immunoregulatory crosstalk between NK cells and APCs has been of great interest, specifically crosstalk between DC and NK cells [14], [15], [16], [21]. However, less attention has been given to reciprocal interaction between NK cells and other APC populations *e.g.*, macrophages that are also important immune effector cells providing immune surveillance against a variety of microbes and tumors [17], [22].

**Figure 8:**
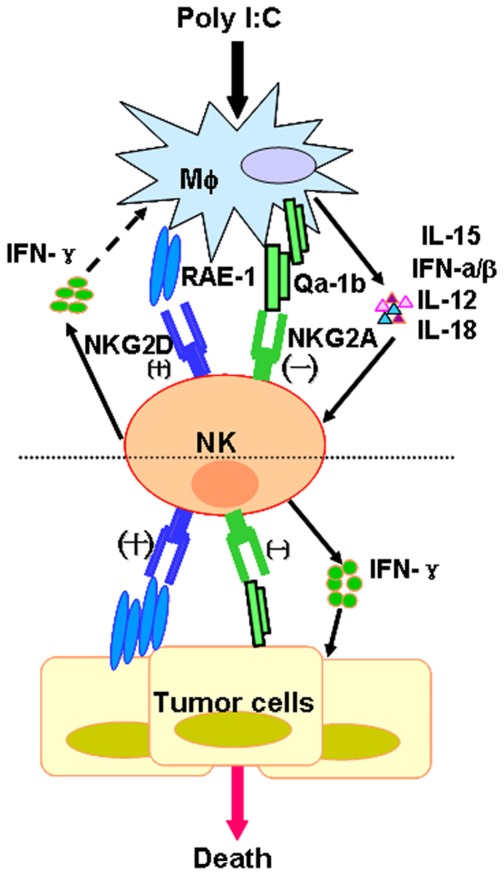
Working model of innate recognition-mediated interaction between NK cells and macrophages during anti-tumor immune response. As a response to stimulation from TLR3 ligand poly I:C, macrophages up-express surface RAE-1 (NKG2D ligand) and produce soluble IL-12, IL-15, and IFN-β. RAE-1 on surface of macrophages and their cytokines will strongly activate NK cells to produce IFN-γ and up-expression of NKG2D. IFN-γ produced by NK cells will stimulate macrophages in feedback. NK cells after activation will directly attack tumor cells by recognizing tumor’s RAE-1 and indirectly arrest tumor growth by secreting IFN-γ. At the same time, poly I:C-induced up-regulation of NKG2A ligand Qa-1 protects macrophages themselves from cytolysis by NK cells in order to keep the positive feedback loop in the anti-tumor immune response.

Poly I:C is a synthetic, double-stranded RNA TLR3 agonist and a potent stimulator of type I IFN production [28], [29]. In the present study, poly I:C was used to stimulate murine macrophages which resulted in NKG2D-dependent upregulation of NK cell-mediated cytotoxicity to susceptible target, which suggests a role for TLR3 signaling in the activation of NK cells upon the interaction between NKG2D and its ligands. We demonstrated a poly I:C-dependent upregulation of NKG2D ligands on macrophages and the contribution of poly I:C-treated macrophages to NK cell lysis against tumor cells in both NKG2D- and cytokine-dependent manner. In accordance with our report, IFN-γ production by NK cells was shown to be cell to cell contact dependent through RAE-1-NKG2D interaction with macrophages in response to B. anthracis spores [22]. Moreover, other TLR agonists (*e.g.* LPS), cytokines (*e.g.* IFN-α, IL-15) or parasite infection, stimulated macrophages or DCs were also found to activate NK cells by increasing expression of NKG2D ligands, and thus augment NK cell-mediated cytotoxicity to tumor or infected cells [19], [30], [31], [32], [33]. In addition, other NK activating receptor may also contribute to lysis of NK cells during the interaction with macrophages. NCR1 (murine NKp46) was reported to be important for NK cell activation during the interaction with S. pneumoniae-infected BMMQ [34]. In human, IFN-γ production of NK cell was shown require the interaction of DNAM-1 and 2B4 with their ligands on macrophage exposure to LPS or bacillus Calmette–Guérin [35]. And NK cell degranulation triggered by interaction with human cytomegalovirus-infected macrophage was inhibited by specific anti-NKp46, anti-DNAM-1, and anti-2B4 mAb [36].

Interestingly, poly I:C-treated macrophages increased NK cell-mediated cytotoxicity against tumor cell, but the macrophages themselves were not killed by NK cells although they exerts increased level of NKG2D ligands expression. These results are in agreement with the recent report that activated NK cells exhibited cytolytic properties against tumor targets but not the infected host macrophage [37]. After comparing the expression levels of ligands for NKG2A and NKG2D between macrophages and YAC-1 targets, we found that a predominant inhibitory signal resulting from NKG2A-ligation over a NKG2D stimulating signal might contribute to the non-sensitive response of macrophages to NK cytolysis. Therefore, we proposed that although poly I:C treatment increased expression of NKG2D ligands on macrophages, signaling via NKG2D receptor was incapable of overriding Qa-1-derived inhibitory signals, and finally can not lead to cytolysis of themselves by NK cells. As for YAC-1 target, the higher expression level of activating ligands and lower expression level of inhibitory ligands might results in predominant activating signaling when contacted with NK cells. In line with the role of inhibitory ligands, it was documented that the inhibitory ligand HLA-E played an important role in the interaction between polyI:C/IFN-γ activated DC and NK cells [21]. The infected proinflammatory (M1) macrophages with high expression of HLA, were more resistant to lysis of NK cells than undifferentiated (M0) and antinflammatory (M2) macrophages [35], [36]. In our experimental system, we hypothesized that these macrophages may be polarized toward M1-like macrophages, as these cells could secrete high level of IL-12 that was a phenotypic character of M1 macrophages. To our knowledge, this is the first report to declare that the preferential expression of inhibitory ligand Qa-1 protected macrophages from cytolysis of NK cells, although other researches have also reported that Qa-1 plays critical role in protection of mature dendritic cells, activated CD4^+^ T cells, or iNKT cells from NK cell-mediated killing [11], [23], [27], [38], [39].

Although NKG2D ligation results in NK cell activation and provides CD8^+^T cell costimulation, it has been reported that sustained binding of NKG2D by its ligands might impair surface expression of functional NKG2D by NK and T cells i.e., prolonged exposure to ligands may desensitize NKG2D on NK cells and T cells, rendering these cells functionally anergic [40], [41], [42]. Hamerman also reported that LPS or Listeria monocytogenes-treated macrophages reduced NKG2D levels on NK cells both *in vitro* and *in vivo* [30]. However, in the present study, we did not find the down-regulation of the NKG2D receptor on NK cells following coculture with poly I:C-treated macrophages. In contrast, we observed that NKG2D expression was markedly increased on splenic NK cells upon exposure to poly I:C-treated macrophages both at the mRNA and protein levels. It has been documented that some cytokines, such as IL-15, IL-12, IL-2, and IFN-α, upregulated NKG2D expression on NK or CD8^+^ T cells [43], [44], [45], [46]. More importantly, IL-15 and IL-2 treatment overcame ligand-induced NKG2D downregulation, enabling NK cells to exhibit substantially enhanced cytotoxicity against tumor cells [47]. We proposed that poly I:C-induced production of cytokines such as IFN-β, IL-12, and IL-15 by poly I:C-treated macrophages overcame the ligand-induced NKG2D downregulation, since antibodies to IL-15 or IFN-β attenuated the expression of NKG2D on NK cells (Fig. 6C). Wang recently also reported that IFN-γ up-regulated membrane IL-15 expression on monocytes prevented NKG2D down-regulation following ligation to NK cells [48]. Meanwhile, our results also strongly showed that the secretion of IFN-β, IL-15, and IL-18 by macrophages contributed to NK cell activation in collaborate with the interaction of NKG2D and its ligands. And these results support the view that the expression of activation markers and the acquisition of cytotoxicity of NK cell against tumor cells required IL-18 and IL-12 released from macrophages [22], [35].

Based on our results, we propose that there is a cross-talk between NK cells and macrophages, which may play major roles in defense against tumors and infectious pathogens. As illustrated in Figure 8, poly I:C stimulation induces up-regulation of NKG2D ligands and secretion of IL-15, IL-12, or IFN-β by macrophages. The interaction of NKG2D and NKG2D ligands as well as these cytokines contribute to NK cell activation. Then the activated NK cells recognize tumor cells by NKG2D, followed by increased secretion of cytotoxic molecules and cytokine IFN-γ, leading to attacking tumors. On the other hand, NKG2A ligand Qa-1 protects macrophages themselves from cytolysis by NK cells. We concluded that the interactions between NK cells and macrophages contributed to anti-tumor immune responses and the innate recognition-mediated crosstalk plays a critical role in this process.

## Supporting Information

Figure S1 **The purity of splenic NK cells isolated from mixed culture cells.** NK cells were first co-cultured with macrophages, and then were separated from macrophages. The purity was determined by flow cytometry. The data are expressed at least three separate experiments.
(TIF)

Figure S2 **NKG2A contributes to the protection of macrophages from NK cell-mediated lysis.** Following transfection with either pan-Qa-1 siRNA (si-Qa-1) or si-control complexed with lipofectamine 2000 at a concentration of 100 nM for 24 h, expression of Qa-1a and Qa-1b in RAW264.7 cells was tested by RT-PCR (A). The data are expressed as the fold change in mRNA expression normalized to untreated cells. * p<0.01 compared to the si-control treatment group. NK cells were isolated after exposed to poly I:C-treated or untreated macrophages and the expression of NKG2A were determined by flow cytometry. Data are expressed as the percentage of positively-stained cells (B). Freshly purified splenic NK cells were first co-cultured with untreated or poly I:C-pretreated macrophages for 24 h at an NK/macrophage ratio of 5∶1, isolated and were then transfected with si-NKG2A or si-control, expression of NKG2A in NK cells was tested by RT-PCR (C), the cytolytic activity of NK cell-mediated lysis was assessed by the ^51^Cr release assay (D). Data are expressed as the mean ± SD from at least three separate experiments. * p<0.01 compared to the si-control or isotype control group using the paired Student’s test.
(TIF)
