# Supplementary figures and images for: Macrophages Help NK Cells to Attack Tumor Cells by Stimulatory NKG2D Ligand but Protect Themselves from NK Killing by Inhibitory Ligand Qa-1
Source: PLoS One. 2012 May 18;7(5):e36928. doi: 10.1371/journal.pone.0036928 (PMC3356357; doi:10.1371/journal.pone.0036928)

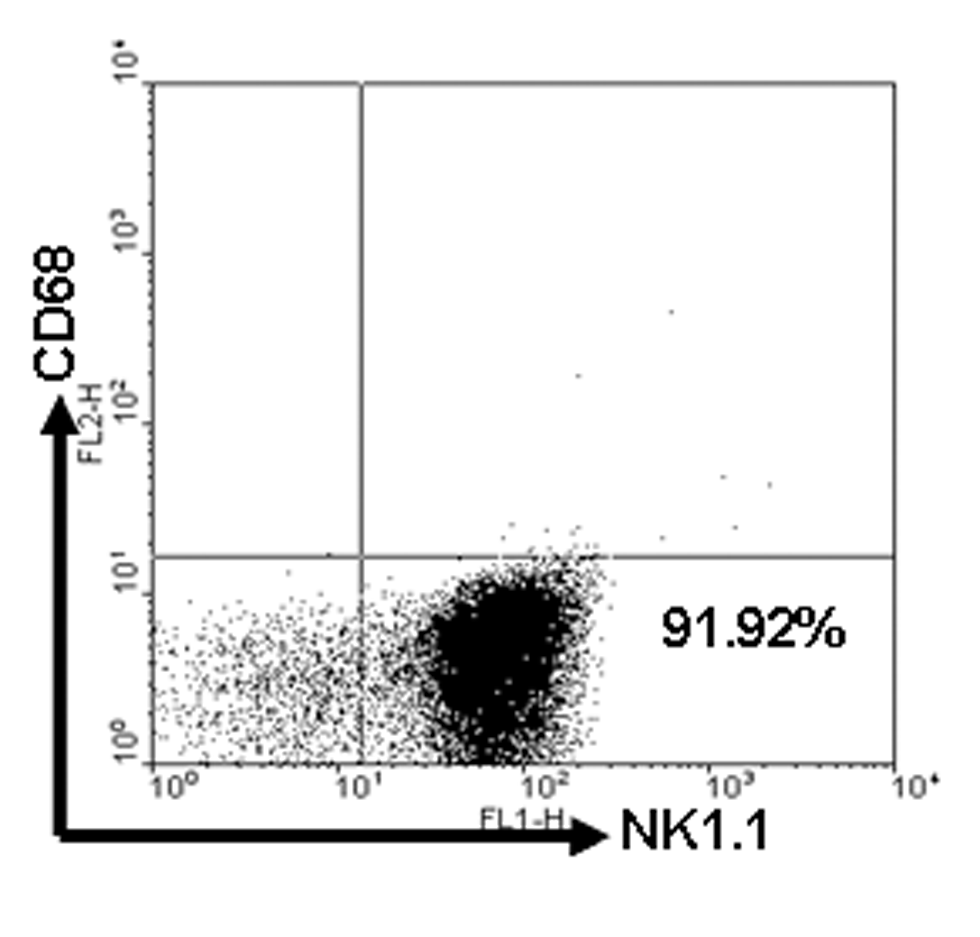

Supplement: Figure S1 — The purity of splenic NK cells isolated from mixed culture cells. NK cells were first co-cultured with macrophages, and then were separated from macrophages. The purity was determined by flow cytometry. The data are expressed at least three separate experiments. (TIF) [file pone.0036928.s001.tif]

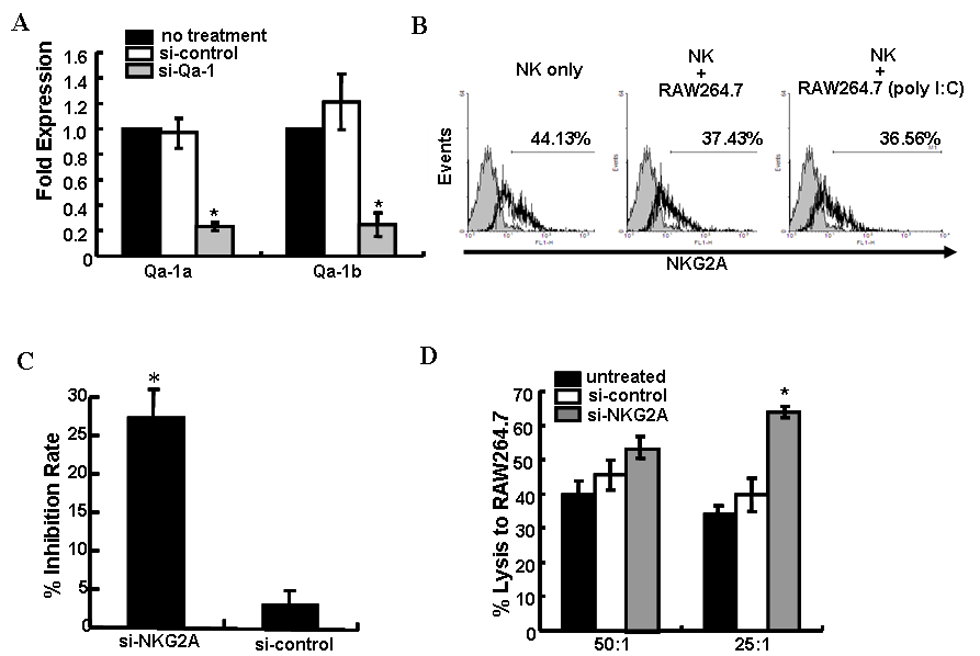

Supplement: Figure S2 — NKG2A contributes to the protection of macrophages from NK cell-mediated lysis. Following transfection with either pan-Qa-1 siRNA (si-Qa-1) or si-control complexed with lipofectamine 2000 at a concentration of 100 nM for 24 h, expression of Qa-1a and Qa-1b in RAW264.7 cells was tested by RT-PCR (A). The data are expressed as the fold change in mRNA expression normalized to untreated cells. * p<0.01 compared to the si-control treatment group. NK cells were isolated after exposed to poly I:C-treated or untreated macrophages and the expression of NKG2A were determined by flow cytometry. Data are expressed as the percentage of positively-stained cells (B). Freshly purified splenic NK cells were first co-cultured with untreated or poly I:C-pretreated macrophages for 24 h at an NK/macrophage ratio of 5∶1, isolated and were then transfected with si-NKG2A or si-control, expression of NKG2A in NK cells was tested by RT-PCR (C), the cytolytic activity of NK cell-mediated lysis was assessed by the 51Cr release assay (D). Data are expressed as the mean ± SD from at least three separate experiments. * p<0.01 compared to the si-control or isotype control group using the paired Student’s test. (TIF) [file pone.0036928.s002.tif]
